# Identification of immunologic and clinical characteristics that predict inflammatory response to C. Novyi-NT bacteriolytic immunotherapy

**DOI:** 10.1186/s12917-018-1424-1

**Published:** 2018-04-02

**Authors:** Amy E. DeClue, Sandra M. Axiak-Bechtel, Yan Zhang, Saurabh Saha, Linping Zhang, David D. Tung, Jeffrey N. Bryan

**Affiliations:** 1 Department of Veterinary Medicine and Surgery, Comparative Internal Medicine Laboratory, University of Missouri, College of Veterinary Medicine, 900 E. Campus Dr, Columbia, MO 65203 USA; 2 Department of Veterinary Medicine and Surgery, Comparative Oncology Radiobiology and Epigenetics Laboratory, University of Missouri, College of Veterinary Medicine, 900 E. Campus Dr, Columbia, MO 65203 USA; 3 Biomed Valley Discoveries, 4435 Main Street, Suite 550, Kansas City, MO 64111 USA

**Keywords:** Immunology, Cancer, Canine, Immunotherapy

## Abstract

**Background:**

Clostridium novyi-NT (CNV-NT), has shown promise as a bacterolytic therapy for solid tumors in mouse models and in dogs with naturally developing neoplasia. Factors that impact the immunologic response to therapy are largely unknown. The goal of this pilot study was to determine if plasma immune biomarkers, immune cell function, peripheral blood cytological composition and tumor characteristics including evaluation of a PET imaging surrogate of tumor tissue hypoxia could predict which dogs with naturally developing naïve neoplasia would develop an inflammatory response to CNV-NT.

**Results:**

Dogs that developed an inflammatory response to CNV-NT had a higher heart rate, larger gross tumor volume, greater tumor [^64^Cu]ATSM SUV_Max_, increased constitutive leukocyte IL-10 production, more robust NK cell-like function and greater peripheral blood lymphocyte counts compared to dogs that did not develop an inflammatory response to CNV-NT. Of these, unstimulated leukocyte IL-10 production, heart rate, and gross tumor volume appeared to be the best predictors of which dogs will develop an inflammatory response to CNV-NT.

**Conclusions:**

Development of inflammation in response to CNV-NT is best predicted by pretreatment unstimulated leukocyte IL-10 production, heart rate, and gross tumor volume.

## Background

For centuries it has been recognized that certain bacterial infections could induce tumor regression. In more modern times, the controlled use of bacteriolytic immunotherapy has been evaluated in both induced and spontaneous tumor models [1]. One such proposed bacterolytic immunotherapy, Clostridium novyi-NT (CNV-NT), has shown promise as a therapy for solid tumors [2, 3]. The advantage of CNV-NT over other types of bacterolytic immunotherapy is that CNV-NT is a strict anaerobe and thus is restricted to growing in the relatively hypoxic regions of tumor tissue and not in healthy tissues. This should, theoretically, maximize local immune stimulation and inflammation in the tumor microenvironment while minimizing systemic effects [4].

C. novyi-NT bacteriolytic immunotherapy has successfully cured induced neoplasia in mouse models and resulted in objective tumor responses in naturally developing neoplasia in the dog [1–3]. However, the response rate in dogs with naturally developing neoplasia has been less than 40%, thus strategies to optimize response rates are needed [2]. Proposed explanations for the limited response rate include a lack of appropriate immune response to infection or variability in the degree of hypoxic tissue in the tumor leading to reduced CNV-NT germination. Dogs with various forms of neoplasia have altered immune function which could impact the efficacy of immunotherapies like induced infection and little is known about the regional blood flow distribution in solid tumors in this species [5–8].

The purpose of this pilot investigation is to identify possible immunologic, tumor or clinical characteristics pre-treatment that would predict which dogs are more likely to successfully develop immunologically pertinent inflammation after administration of CNV-NT. To evaluate this, plasma immune biomarkers, immune cell function, peripheral blood cytological composition and tumor characteristics including evaluation of a PET imaging surrogate of tumor tissue hypoxia were measured in dogs with naturally developing naïve neoplasia prior to administration of CNV-NT; following CNV-NT treatment the development of inflammation was recorded. Baseline parameters were compared between dogs that developed inflammation and those that did not. Parameters that were significantly different between groups were then used to develop a predictive model.

## Methods

### Population

Client owned dogs presented to the University of Missouri Veterinary Health Center with histologically or cytologically confirmed soft-tissue sarcoma, oral or cutaneous malignant melanoma, oral squamous cell carcinoma, or other cutaneous carcinomas were eligible for enrollment with written informed client consent (IACUC protocol #7386). Eligibility criteria included tumors greater than 2 cm in diameter, tumor size and location where surgical excision with at least marginal resection was a viable option, and body weight of > 10 kg. Dogs were excluded if comorbidities were present that suggested survival expectation of less than 6 weeks, evidence of metastasis outside of the local draining lymph node, tumor location where abscess development would be catastrophic (e.g., CNS), persistent neutropenia or thrombocytopenia, grade 2 increases in plasma ALT, BUN or creatinine, administration of antimicrobial therapy within the 7 days preceding enrollment, concurrent infection requiring systemic antimicrobial therapy, chemotherapy, radiation therapy, or other immunotherapy within the 3 weeks preceding enrollment, pregnancy or potential pregnancy, enrollment in another clinical trial, cardiac disease severe enough that a balanced crystalloid solution administered at 4 mL/kg/h would likely induce congestive heart failure, and unavailability during the full study duration.

Baseline evaluations included medical history, physical examination, complete blood count, plasma biochemical profile, complete urinalysis, thoracic radiographs, and cytologic evaluation of the draining lymph bed if accessible. Prior to therapy, tumors were evaluated using PET/CT to evaluate tumor size, estimate glucose metabolism with [^18^F]FDG, and estimate relative tumor hypoxia using [^64^Cu]ATSM (some data not presented in this manuscript).

#### Treatments

Dogs received CNV-NT (BioMed Valley Discoveries Inc., Kansas City, MO) either intravenous (IV) or intratumoral (IT) on day 0. The change from IV to IT was based on a programmatic decision by the sponsor.

##### Intravenous administration

CNV-NT (1 × 10^9^) spores were administered in 50 mls of physiologic saline through an intravenous catheter. Initially, 5 mL was administered over 2 min; dogs’ vital parameters were monitored for 30 min. If the dog’s vital parameters were within 10% of baseline after 30 min, the remaining 45 mL was administered over a 5 min time period. Following completion of CNV-NT infusion, intravenous fluids were administered for 2 h and dogs were discharged to their owners if no signs of a reaction was noted.

##### Intratumoral administration

CNV-NT was provided in 3 mL of saline at a dose of 1 × 10^8^ spores. Following sedation, the tumor was clipped and aseptically prepped, and CNV-NT was administered directly into the tumor with 0.75 mL injected into 4 total sites evenly distributed within the tumor. Vital parameters were monitored for 3 h following injection.

#### Groups

The primary outcome measure used to group the dogs was the development of clinically identifiable inflammation within 28 days of treatment for the IV group and within 56 days of treatment for the IT group. In the IV CNV-NT group, dogs that developed fever (rectal temperature > 39.2 °C (102.5 °F)) and lethargy were placed in the inflammation group while dogs that developed no clinical signs following infusion were classified as having not developed inflammation. In the IT group, dogs that developed clinical evidence of abscess formation including edema, erythema, and/ or mucopurulent discharge were placed in the inflammation group. Dogs that had no evidence of abscess formation were placed in the no evidence of inflammation group.

### Sample collection and processing

At baseline (pre-treatment), blood was collected from the jugular vein and placed in either EDTA, sodium heparin, lithium heparin or evacuated tubes for immunologic evaluation. Lithium heparinized blood was immediately cooled and centrifuged within 1 hour of collection. Plasma or serum was collected and stored at − 80 °C for batch analyses of immune protein evaluation. The remainder of the blood was processed immediately for PBMC isolation, CBC, phagocytic function and leukocyte cytokine production assays as indicated below.

### Clinical/tumor characteristics

Dogs were evaluated prior to therapy utilizing rectal temperature, heart rate, complete blood count, plasma globulin concentration (total protein- albumin = globulin), CT scan measurement of tumor volume and PET scan with [^18^F]FDG to characterize tumor metabolism and [^64^Cu]ATSM to characterize tumor hypoxia. [^64^Cu]ATSM is reduced and trapped in hypoxic cells, localizing for imaging with positron emission tomography (PET). Dogs were injected with 74 MBq (2 mCi) of [^64^Cu]ATSM 45 min prior to scanning. Dogs were anesthetized and maintained on a propofol infusion breathing room air. Scans were acquired with a Philips C-PET+ with attenuation correction. Reconstructed images were evaluated using MIMfusion software. Maximum standard uptake value (SUV_Max_) and threshold volume with SUV > 1.0 were calculated.

#### Plasma immune biomarkers

Plasma was collected centrifuged at 400 g for 15 min and then stored at − 80 °C for batch analysis. Thirteen immune markers: IL-6, CXCL-8, IL-2, IL-7, TNF-α, GM-CSF, IL-18, CXCL-1, IP-10, IL-15, IL-10 and MCP-1 were evaluated in undiluted plasma as previously described using canine specific multiplex bead-based, ELISA assay (Millipore Sigma, Burlington, MA), a MAGPIX Multiplexing instrument and analyzed using MILLPLEX Analyst 5.1 software [9]. Serum HMGB-1 (IBL International, Toronto, ON) and CRP (ICL Inc., Portland OR) were measured using commercially available ELISAs according to the manufacturers' recommendations [10, 11].

#### Immune cell function

Immune cell function was assessed by evaluating granulocyte phagocytic and respiratory burst function, leukocyte cytokine production capacity and NK-like cell function.

Phagocytic function was determined using the Phagotest® commercial test kit (Orpegen Pharma, Heidelberg, Germany) which evaluates phagocytosis of FITC-labeled, opsonized *E. coli.* The assay has been previously validated in dogs and was performed as previously described [5, 7]. Samples were analyzed by flow cytometry using the CyAn ADP flow cytometer (Beckman Coulter, Brea, CA) and associated data analysis software (Summit V 5.2.0.7477, Brea, CA) within 30 min and a minimum of 15,000 events were recorded for each sample. DNA stain positive cells were gated and placed on a forward and side scat plot. Phagocytes were identified using standard forward and side scatter characteristics. Then, FITC positive phagocytes were identified on a histogram. Both the relative number of *E. coli*-positive cells as well as the mean fluorescence intensity (MFI) of positive cells indicating the number of bacteria per cell were recorded.

The quantification of oxidative burst of PMNs was performed using a Phagoburst® kit (ORPEGEN Pharma, Heidelberg, Germany) which evaluates *E.coli* and PMA–induced oxidative burst using dihydrorhodamine 123 as a fluorogenic substrate. The assay has been previously validated in dogs and was performed as previously described [5, 7]. Samples were analyzed by flow cytometry using the CyAn ADP flow cytometer and associated data analysis software within 30 min and a minimum of 15,000 events were recorded for each sample. DNA stain positive cells were gated and placed on a forward and side scatter plot. Phagocytes were identified using standard forward and side scatter characteristics. Then, a FLI histogram was used to identify positive phagocytes. The percentage of positive cells indicating recruitment and the MFI indicating the intensity of oxidative burst were recorded.

Leukocyte cytokine production capacity was determined by stimulating whole blood with lipopolysaccharide (LPS) from *Escherichia coli* 0127:B8 (final concentration, 100 ng mL^− 1^; Sigma-Aldrich, St. Louis, MO), lipoteichoic acid (LTA) from *Streptococcus faecalis* (final concentration, 1000 ng mL^− 1^; Sigma-Aldrich), or phosphate buffered saline (PBS; unstimulated control) and then measuring cytokine concentrations in the cell culture supernatant as previously described [12]. Blood was diluted 1:2 with media and samples were cultured on 12 well plates with LPS, LTA or PBS and then incubated for 24 h at 37 °C in 5% CO_2_. Cell supernatant was collected at end of incubation and stored in − 80 °C for analysis. Quantification of TNF-α, IL-6 and IL-10 was accomplished using a canine specific multiplex bead-based, ELISA assay (Millipore Sigma) and a MAGPIX Multiplexing instrument as stated for the plasma immune markers.

NK-like cell function was determined using a thyroid adenocarcinoma cytotoxicity assay as previously described [8]. Canine thyroid adenocarcinoma (CTAC) cells were used as target cells. Prior to the assay, CTAC cells were labeled with 3 mM green fluorescent 3,3′-Dioctadecyloxacarbocyanine (DiO) for 20 min at 37 °C in 5% CO_2_. Cytotoxicity of cancer cells was assessed by co-incubating PBMC with DiO-labeled CTAC cells for 24 h at 37 °C with 5% CO_2_. Cells were comingled in different PBMC to Dio-CTAC cell ratios: 1:1, 10:1, 25:1 and 50:1. Single cell population of PBMC or Dio-CTAC were used as controls. At end of incubation, cells were incubated with propidium iodide (PI). Samples were analyzed using the CyAn ADP flow cytometer and associated data analysis software. A minimum of 10,000 events were recorded for each sample. Data were analyzed as previously described [8]. Briefly, the CTAC cells were gaited on a forward/side scatter plot and then applied to a plot comparing DiO and PI. DiO and PI positivity were determined using unstained cells as controls. Cells positive for DiO and PI were defined as dead CTAC cells. Baseline cell death was established using DiO/PI stained CTAC cells alone. The NK-like cell killing index was calculated by dividing the % death from the PBMC+CTAC cell mixture by the CTAC cells alone.

#### Peripheral blood immune cell composition

**Fig. 1:**
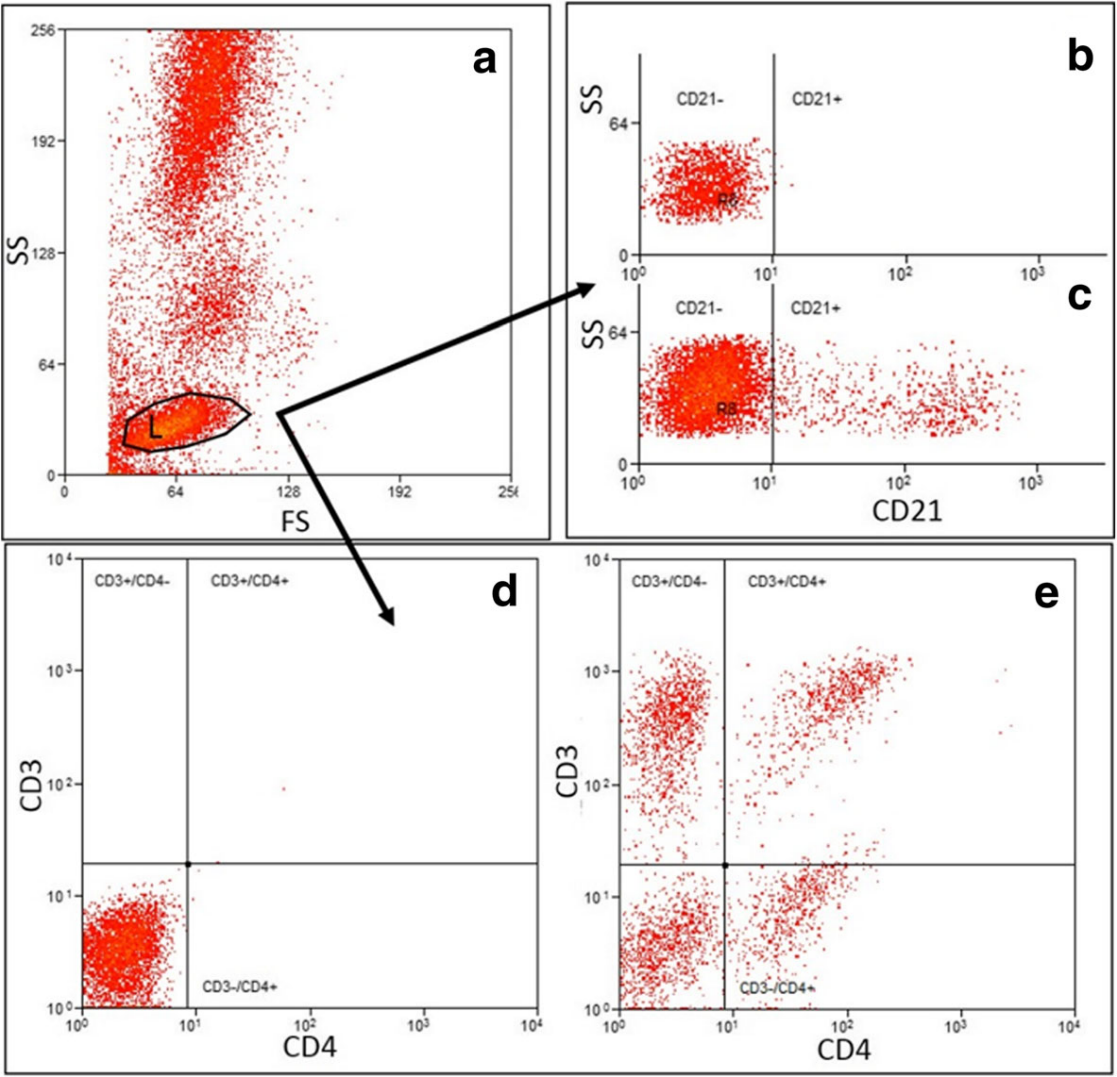
Example gating scheme for identification of CD3^+^/CD4^+^ lymphocytes and CD21^+^ lymphocytes. Lymphocytes were identified and gated using a forward and side scatter plot (**a**). Gated lymphocytes were applied were then applied to PE (CD3) vs FITC (CD4) plots or an Alexa Fluor 647 (CD21) histogram, respectively. Unstained cells and matched isotype controls from the same manufacturer were used to determine cut offs for negative (**b** and **d**) versus positive (**c** and **e**) cells. CD3^+^/CD4^+^ or CD21 positive cells were then identified. CD3^+^/CD8^+^ cells were selected in the same manner as the CD3^+^/CD4^+^ cells

**Fig. 2:**
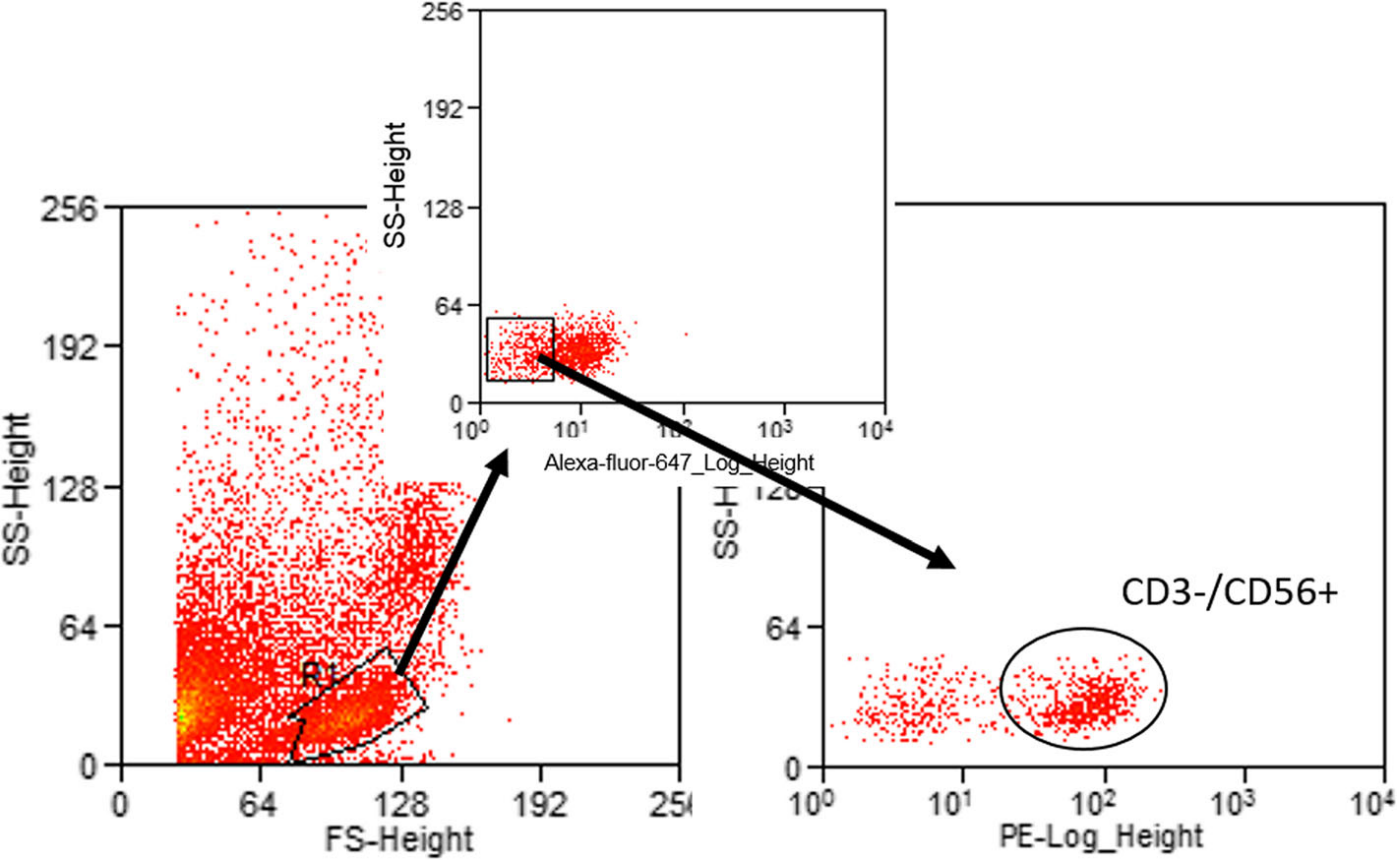
Example gating scheme for identification of CD3-/CD56+ lymphocytes. Lymphocytes were identified and gated using a forward and side scatter plot. Gated lymphocytes were applied to an Alexa Fluor 647 (CD3) vs side scatter plot and selecting for the Alexa Fluor 647 negative cells. Then PE positive cells were selected on a PE (CD56) vs side scat plot

Peripheral blood immune cell composition was determined by performing a complete blood count to determine the number of total white blood cells, neutrophils, monocytes and lymphocytes in the peripheral blood and flow cytometry to determine lymphocyte phenotype. A complete blood count was performed by the University of Missouri Veterinary Diagnostic Laboratory. Antibodies used for PBMC phenotype assay were rat anti-mouse CD3-PE (abcam, Cambridge, UK; Clone KT3), mouse anti-dog CD21-Alexa Fluor 647 (AbD Serotec, Raleigh, NC; Clone: CA2.1D6), rat anti-mouse/rat FoxP3-APC (eBioscience, San Diego, CA; Clone: FJK-16 s), rat-anti-dog CD4-FITC (AbD Serotec; Clone: YKIX302.9), mouse anti-human CD25-PE (Dako; Clone: ACT-1), rat anti-human CD3-Alexa Fluor 647 (AbD Serotec; Clone: CD3-12), rat anti-dog CD8-APC (eBioscience; Clone: YCATE55.9), anti-human CD56-PE (Dako, Santa Clara, CA; Clone: MOC-1), Rat IgG2a APC isotype control, Mouse IgG1 PE isotype control and Rat IgG2a FITC isotype control (eBioscience). PBMCs were harvested and 200 μl of 10^6^ PBMC per sample were added to a 96 well round bottom plate. Plates were centrifuged at 300 g for 6 min and supernatant removed. 50 μl of FACS buffer was added to each sample and then samples were stained for the following markers: CD4 T cell (CD3^+^/CD4^+^), CD8 T cell (CD3^+^/CD8^+^), NK-like cell (CD3^−^/CD56^+^) and B cell (CD21^+^) for 30 min on ice protected from light. At end of incubation cells were washed twice with PBS and fixed with 2% paraformaldehyde. T regulatory cells (CD4^+^/CD25^+^/FoxP3^+^) were detected using surface CD4 and CD25 stains and incubating for 30 min as previously described [13]. Cells were washed with PBS, then fixed and permeabilized with fix/perm FoxP3 staining buffer (eBioscience) for 30 min to detect intracellular FoxP3. At the end of incubations, cell were washed and collected into FACS tubes; all samples were then analyzed at the University of Missouri Cell and Immunology Core Facility using CyAn ADP and Summit software (Summit V 5.2.0.7477, CA, USA). A minimum of 15,000 events were recorded for each sample. Unstained cells and matched isotype controls from the same manufacturer were used for negative antibody controls for analyses. Lymphocytes were identified and gated using a forward and side scatter plot. To evaluate CD3^+^/CD4^+^ or CD3^+^/CD8^+^ T cells, the gated lymphocytes were then applied to PE (CD3) vs FITC (CD4) or PE (CD3) vs APC (CD8) plots, respectively, and double positive cells were identified (Fig. 1). To identify B cells, lymphocytes were applied to an Alexa Fluor 647 (CD21) histogram (Fig. 1). NK-like cells were identified by applying the gated lymphocytes to an Alexa Fluor 647 (CD3) vs side scatter plot and selecting for the Alexa Fluor 647 negative cells. The CD3 negative cells were then applied to a PE (CD56) vs side scat plot and the PE positive cells were selected (Fig. 2). T regulatory cells were identified by applying the gated lymphocytes to a FITC (CD4) vs PE (CD25). Then FITC and PE double positive cells were applied to an APC (Fox P3) vs side scatter plot as previously described [13].

### Statistical analysis

Statistical analysis was accomplished using SigmaPlot software (SigmaStat; Systat Software Inc., San Jose, CA). To construct the initial model, variables were compared using a univariate analysis to identify parameters that differed between dogs that developed inflammation and those that did not. A Mann-Whitney Rank Sum test was used in initial comparison of variables between groups. Because the goal of this pilot investigation was to identify markers that would predict which dogs developed inflammation at the tumor site and we did not want to inappropriately exclude markers due to type II statistical error, a *p*-value of < 0.10 was considered statistically significant and variables with a *p*-value less than this cut-off were included in predictive modeling. Parameters that were significantly associated with the development of inflammation were then entered into a best subsets regression model. Best subsets were developed based on R squared as the best criterion. The final model selection was determined by selecting the model with the greatest R squared with the smallest Mallows Cp value, significant contribution from all variables (*p* < 0.05) and minimal multicollinearity based on a variance inflation factor of < 1.50. Data are presented as median and range unless otherwise noted.

## Results

### Patient population

Twenty dogs were enrolled, 10 dogs received IV and 10 dogs received IT administration of CNV-NT. The median age of dogs enrolled was 9.5 years (range 5-13 years, age of one dog unknown) and the median weight was 28.2 kg (range 14.4-70 kg). The sexes included intact male (*n* = 2), neutered male (*n* = 10), and spayed female (*n* = 8). Breeds of dogs enrolled were Labrador retriever (*n* = 5), Golden retriever (*n* = 3), Husky (*n* = 3), and one each of the following breeds: Standard Poodle, American Staffordshire terrier, English setter, Pug, Beagle, Bloodhound, Border Collie, Pointer, and mixed breed. Tumor types were soft tissue sarcoma (STSA) (*n* = 10), oral sarcoma (*n* = 6), oral melanoma (*n* = 3), and carcinoma (*n* = 1). One dog in the IV group was excluded from analysis due to development of gastric dilation and volvulus 9 days post-infusion, necessitating surgery and subsequent antibiotic therapy. This dog was withdrawn from trial and 19 dogs were included in the immunologic analysis. Of the dogs with evaluable data, 14 were classified as having developed inflammation, six in the IV group and eight in the IT group; 5 did not develop inflammation. There was no difference in body weight between the groups.

### Clinical/Tumor parameters

**Fig. 3:**
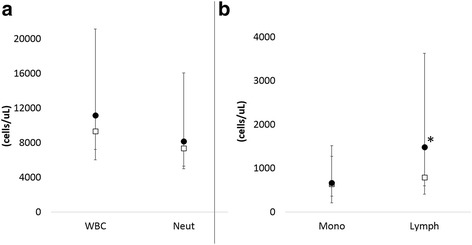
Comparison of median and range of (**a**) peripheral blood white blood cell (WBC), neutrophil (Neut), (**b**) monocyte (Mono) and  lymphocyte (Lymph) counts between dogs that developed inflammation (closed circles) and those that did not (open squares). *p = 0.087

**Fig. 4:**
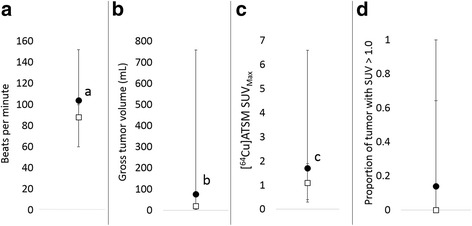
Comparison of median and range of heart rate (**a**), gross tumor volume (**b**), [^64^Cu]ATSM SUV_Max_ (**c**) and threshold volume of tissue with SUV > 1.0 (**d**) between dogs that developed inflammation (closed circles) and those that did not (open squares). ^a^*p* = 0.055. ^b^*p* = 0.058, ^c^*p* = 0.092

Peripheral blood white blood cell, neutrophil and monocyte counts did not differ between groups (Fig. 3). However, peripheral blood lymphocyte count was significantly greater in the dogs that developed inflammation compared to those that did not (*p* = 0.087). Heart rate was significantly higher in dogs that developed inflammation compared to those that did not (Fig. 4, *p* = 0.055). Gross tumor volume was significantly larger for dogs that developed inflammation compared to those that did not (Fig. 4, *p* = 0.058). [^64^Cu]ATSM scan SUV_Max_ was significantly greater for dogs that developed inflammation (Fig. 4, *p* = 0.092), but there was no difference in the threshold volume of tissue with SUV > 1.0 between groups. There was no difference in initial rectal temperature between groups (data not shown).

### Serum/plasma immune biomarkers

**Fig. 5:**
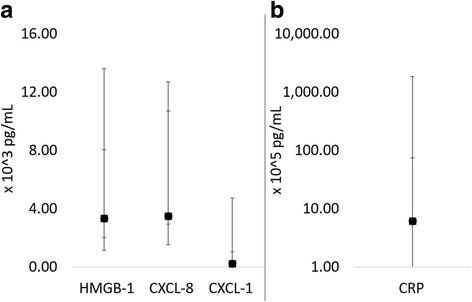
Comparison of median and range of serum (**a**) HMGB-1 and (**b**) CRP and plasma (**a**) CXCL-8 and (**a**) CXCL-1 concentrations between dogs that developed inflammation (closed circles) and those that did not (open squares). Please note that CRP is on a log scale.   There were no significant differences between groups

Plasma concentrations of IL-6, IL-2, IL-7, GM-CSF, IL-18, IL-15, MCP-1, TNF-α, IL-10 and IP-10 fell below the lower limit of detection for ≥50% of the samples and thus were not statistically analyzed (data not shown). Serum HMGB-1 and CRP and plasma CXCL-8 and CXCL-1 concentrations were not significantly different between groups (Fig. 5).

### Immune cell function

**Fig. 6:**
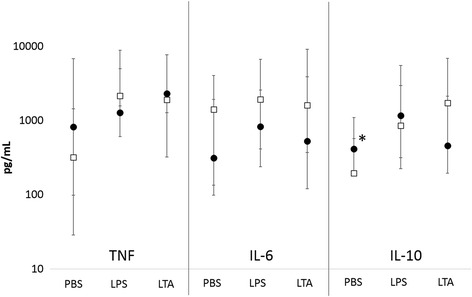
Comparison of median and range for PBS (control, unstimulated), lipopolysaccharide (LPS) and lipoteichoic acid (LTA) stimulated TNF-α, IL-6 and IL-10 on a logarithmic scale between dogs that developed inflammation (closed circles) and those that did not (open squares). *p = 0.060

**Fig. 7:**
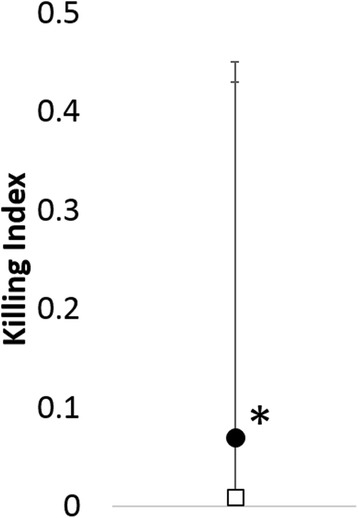
Comparison of median and range for NK-like cell function killing index between dogs that developed inflammation (closed circles) and those that did not (open squares). *p = 0.043

**Fig. 8:**
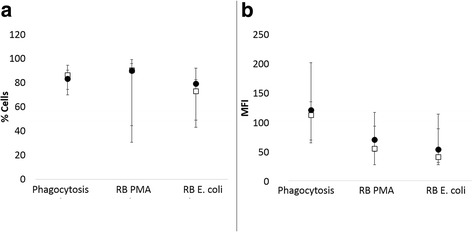
Comparison of median and range of the percentage of cells (**a**) undergoing phagocytosis of opsonized *E. coli* (phagocytosis), PMA-induced respiratory burst (RB PMA) or *E. coli*-induced respiratory burst (RB *E. coli*) or the (**b**) number of *E. coli* being phagocytized or the intensity of respiratory burst between dogs that developed inflammation (closed circles) and those that did not (open squares). There were no significant differences between groups

Unstimulated leukocyte IL-10 (Fig. 6, *p* = 0.060) production was significantly greater in the dogs that developed an inflammatory response compared to those that did not. Unstimulated leukocyte TNF-α and IL-6 production and LPS or LTA-stimulated leukocyte TNF-α, IL-6 and IL-10 production did not significantly differ between groups (Fig. 6). NK cell function was significantly greater in the dogs that developed an immune response compared to those that did not (Fig. 7, *p* = 0.043). There was no difference in the percentage of cells undergoing phagocytosis or the number of bacteria phagocytized, nor was there a difference in the percentage of cells performing oxidative burst stimulated by *E. coli* or PMA or the intensity of the oxidative burst between groups (Fig. 8).

### Peripheral lymphocyte composition

**Fig. 9:**
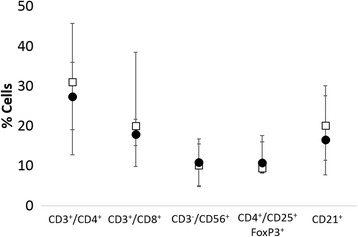
Comparison of median and range of peripheral, circulating lymphocyte phenotype [CD4 T cell (CD3^+^/CD4^+^), CD8 T cell (CD3^+^/CD8^+^), NK-like cell (CD3^−^/CD56^+^) T regulatory cells (CD4^+^/CD25^+^/FoxP3^+^) and B cell (CD21^+^)] between dogs that developed inflammation (closed circles) and those that did not (open squares). There were no significant differences between groups

While peripheral blood lymphocyte count was significantly greater in the dogs that developed inflammation compared to those that did not, there were no differences in peripheral blood lymphocyte phenotype (CD4, CD8, CD21, Treg) between groups (Fig. 9).

### Predicting inflammation

Best subsets regression was used to evaluate the variables unstimulated leukocyte IL-10 production, leukocyte NK-like activity, heart rate, lymphocyte count, gross tumor volume and [^64^Cu]ATSM scan SUV_Max_ max for prediction of inflammation. The model containing a combination of unstimulated leukocyte IL-10 production, heart rate, and gross tumor volume appeared to be the best predictor of inflammation (Adjusted R squared 0.553; Cp 2.405; *p* < 0.034).

## Discussion

Dogs that developed an inflammatory response to CNV-NT had a higher heart rate, larger gross tumor volume, greater tumor [^64^Cu]ATSM SUV_Max_, increased constitutive leukocyte IL-10 production, more robust NK cell-like function and greater peripheral blood lymphocyte counts compared to dogs that did not develop an inflammatory response to CNV-NT. Of these, unstimulated leukocyte IL-10 production, heart rate, and gross tumor volume appeared to be the best predictors of which dogs will develop an inflammatory response to CNV-NT.

Ideally, we would use routinely measured clinical parameters to identify which dogs are more likely to have an inflammatory response to CNV-NT. In this study, we evaluated clinical and tumor characteristics including gross tumor volume, [^64^Cu]ATSM uptake, heart rate, rectal temperature, hematocrit, peripheral white blood cell count and plasma globulin concentrations as possible clinical biomarkers. However, only heart rate, peripheral leukocyte cell count, [^64^Cu]ATSM uptake and gross tumor volume were significantly different between groups. Of these, heart rate and gross tumor volume were predictors of development of inflammation in response to CNV-NT administration in our best subsets model. Tachycardia could be caused by activation of the sympathetic nervous system which has been associated with more severe inflammatory responses to infection. This might explain why dogs that developed inflammation had higher heart rates. It has traditionally been expected that as tumor volume increases, the relative percentage of hypoxic tissue increases. Germination of CNV-NT appears to be more robust in tumors with larger volumes and greater hypoxic regions [14]. Therefore, it was expected that a greater [^64^Cu]ATSM SUV_Max_ and larger tumor volume would be associated with greater germination and a more intense inflammatory response.

Failure to generate an appropriate immune response to infection could impact the efficacy of CNV-NT bacteriolytic immunotherapy. Immunodysfunction has been identified in dogs and people with cancer. Altered neutrophil phagocytosis, respiratory burst function, cytokine production, peripheral circulating lymphocyte phenotype distribution and NK-cell like function have all been reported in dogs and people with cancer [5–8]. Whether these changes represent a paraneoplastic syndrome or primary immunodysfunction is unclear and it is likely that both mechanisms are observed in the dog and human population. In evaluating baseline immune system characteristics, we found that constitutive leukocyte IL-10 production, NK cell-like function and greater peripheral blood lymphocyte counts were significantly different between dogs that developed inflammation in response to CNV-NT and those that did not. Of these, only constitutive leukocyte IL-10 production was selected as a predictor of development of inflammation in response to CNV-NT administration in our best subsets model. This was an unexpected finding since production of IL-10 is an immunosuppressive cytokine that has been implicated in cancer-associated immunodysfunction. It is possible that dogs with greater constitutive IL-10 production were immunosuppressed at the time of CNV-NT administration. This immunosuppression allowed CNV-NT germination to go unchecked by the immune system initially, allowing for a larger bacterial dose than dog with less IL-10 and a competent immune system. This greater bacterial dose eventually provided enough stimulus to overcome the immunosuppression and allow for the measurable inflammatory response.

We identified differences in NK cell-like function and peripheral blood lymphocyte counts between the dogs that developed inflammation and those that did not, however, these characteristics were not identified as good predictors of the development of inflammation. Stress-induced lymphopenia is a common finding in clinically ill dogs. This physiologic response is mediated by glucocorticoids, which also have direct immunosuppressive effects. It is possible that dogs with lymphopenia had greater circulating concentrations of glucocorticoids which resulted in suppression of the inflammatory response to CNV-NT. NK-like cells are important in anti-tumor and cytotoxic immune responses. NK-cells are also thought to be important effector cells during clostridium infection [15]. Greater baseline NK-like cell function might have allowed for a greater immune response to CNV-NT germination. Larger-scale studies focused on the impact of stress hormones and baseline NK-like cell function are needed to tease out the importance of these markers.

There were several limitations to this study. First, this was a pilot study and therefore the number of dogs enrolled was limited and the cut-off point for determining significance was a *p* < 0.10. The rationale for this was to avoid type II statistical error and insure that any possible candidate biomarker would be identified to assist with development of future studies in a larger patient population. We also did not compare difference between IV and IT administration. Prior to clinical use of these biomarkers, they must be studied in a larger population and comparisons made between IV and IT administration. We did not correlate induction of inflammation with tumor response or with definitive germination of CNV-NT in this study, but this is needed to understand the full importance of these biomarkers. Further, while this study focused on solid tumors, there were three histologic tumor types in multiple anatomic locations. It is possible that this heterogeneity impacted our results. The lower limit of detection of the plasma immune markers are relatively high due to the relative analytical insensitivity of the currently available assays for dogs. In the future, more analytically sensitive assays should be developed.

## Conclusion

In this pilot study, we identified several parameters that might help identify dogs with cancer that are more likely to have an inflammatory response to CNV-NT. In the future, investigators should consider evaluating if heart rate, tumor volume, surrogate markers of tumor hypoxia, IL-10 production, NK-like cell function and circulating lymphocyte count could be useful markers for patient selection or stratification of patients in clinical trials evaluating CNV-NT for the treatment of cancer.
